# Selective Antimicrobial Chitosan Films Incorporating Green-Synthesized Silver and Copper Oxide Nanoparticles for Acne Treatment

**DOI:** 10.3390/antibiotics14090891

**Published:** 2025-09-03

**Authors:** Roberta Albino dos Reis, Carolina C. de Freitas, Leonardo Longuini da Silva, Laura Pierobão Monteiro, Gerson Nakazato, Mathilde Champeau, Ricardo A. Galdino da Silva, Amedea Barozzi Seabra

**Affiliations:** 1 Centro de Ciências Naturais e Humanas (CCNH), Universidade Federal do ABC (UFABC), Santo André 09210-580, SP, Brazil; carolina.freitas@ufabc.edu.br (C.C.d.F.); mathilde.champeau@ufabc.edu.br (M.C.); 2 Laboratory of Basic and Applied Bacteriology, Department of Microbiology, Center of Biological Sciences, State University of Londrina, Londrina 86057-970, PR, Brazil; laura.pierobao@uel.br (L.P.M.); gnakazato@uel.br (G.N.); 3 Instituto de Ciências Ambientais, Químicas e Farmacêuticas, Universidade Federal de São Paulo, Diadema 09913-030, SP, Brazil; galdino.ricardo@unifesp.br

**Keywords:** chitosan films, silver nanoparticles, copper oxide nanoparticles, green synthesis, antimicrobial activity, cytotoxicity, *Camellia sinensis*

## Abstract

**Background/Objectives:** Chitosan-based films incorporating green-synthesized silver nanoparticles AgNPs) or copper oxide nanoparticles (CuONPs) were developed to compare their selective antimicrobial action for topical applications. While AgNPs are known for broad-spectrum activity, this study hypothesized that CuONPs would exhibit superior, targeted efficacy against the acne-associated bacterium *Cutibacterium acnes*. **Methods:** Nanoparticles were synthesized using Camellia sinensis extract and characterized. Antimicrobial activity was evaluated using Minimum Inhibitory Concentration (MIC) and Minimum Bactericidal Concentration (MBC) assays. Chitosan films containing AgNPs or CuONPs were further tested for selective antimicrobial activity and fibroblast cytocompatibility. **Results:** AgNPs showed strong activity against *Escherichia coli* and *Staphylococcus aureus* (MIC = 15 µg/mL) but were less effective against *C. acnes* (MIC = 125 µg/mL). In contrast, CuONPs demonstrated selective efficacy against *C. acnes* (MIC = 62 µg/mL; MBC = 125 µg/mL). When incorporated into chitosan films, AgNPs@CHI inhibited *E. coli* (35 mm halo) and *S. aureus* (30 mm), whereas CuONPs@CHI were selectively effective against *C. acnes* (45 mm). All films preserved fibroblast viability above the 70% ISO 10993-5 threshold. **Conclusions:** CuONPs@CHI films validated selective anti-C. acnes performance, highlighting their promise for targeted anti-acne therapies, while AgNPs@CHI films served as effective broad-spectrum antimicrobial barriers.revealed that AgNPs were potent against *Escherichia coli* and *Staphylococcus aureus* (MIC = 15 µg/mL) but less effective against *C. acnes* (MIC = 125 µg/mL). Conversely, CuONPs demonstrated a marked selective advantage against *C. acnes* (MIC = 62 µg/mL; MBC = 125 µg/mL). When incorporated into chitosan films, AgNPs@CHI films inhibited *E. coli* (35 mm halo) and *S. aureus* (30 mm), whereas CuONPs@CHI films were selectively effective only against *C. acnes* (45 mm), confirming the targeted performance. All films maintained fibroblast viability above the 70% ISO 10993-5 cytotoxicity threshold. These findings validate the selective action of CuONPs@CHI films, positioning them as a promising biomaterial for targeted anti-acne therapies, while AgNPs@CHI films serve as effective broad-spectrum antimicrobial barriers.

## 1. Introduction

Chitosan (CHI) is a natural biopolymer derived from chitin, known for its biodegradability, biocompatibility, and intrinsic antimicrobial properties. Its reactive functional groups—such as primary amines (–NH_2_) and hydroxyls (–OH)—enable a wide range of chemical modifications and allow for the chelation of metal ions, enhancing its applicability in biomedical and pharmaceutical fields [1,2]. These characteristics make chitosan particularly attractive for the development of films and coatings aimed at preventing microbial contamination and promoting skin health [1,2,3].

In dermatology, chitosan-based materials have gained growing attention, especially in the treatment of acne vulgaris—a chronic inflammatory condition closely associated with bacterial colonization by *Cutibacterium acnes* and *Staphylococcus aureus* [4]. The increasing prevalence of antibiotic-resistant strains has intensified the need for alternative antimicrobial strategies [4]. In this context, biocomposites formed by chitosan and metal-based nanoparticles (M-NPs), such as silver nanoparticles (AgNPs) and copper oxide nanoparticles (CuONPs), offer a promising solution. These nanoparticles exhibit potent antimicrobial effects by disrupting bacterial membranes, generating reactive oxygen species (ROS), and interfering with key cellular processes, ultimately reducing bacterial viability. AgNPs, in particular, are known for their ability to release Ag^+^ ions and penetrate biofilms, while CuONPs promote oxidative damage through ROS production and direct metal ion interactions with biomolecules [5,6,7,8,9]. The incorporation of these nanoparticles into chitosan matrices enhances the stability and bioavailability of the active agents while allowing for controlled ion release and improved handling [8,10]. When synthesized through green chemistry routes using plant extracts such as *Camellia sinensis* (green tea), these nanoparticles benefit from biocompatible surface capping by phytochemicals—such as flavonoids and polyphenols—which also reduce toxicity and environmental impact [5,10].

The development of such biogenic nanocomposites is particularly important for sustainable dermatological solutions. However, most existing studies focus on single-metal systems or fail to evaluate the effects of these nanocomposites on acne-related pathogens such as *C. acnes*. There is also a lack of systematic comparisons between AgNP- and CuONP-based films synthesized through truly green routes, especially in terms of their antimicrobial spectrum and cytotoxic selectivity. In this context, the present study addresses these gaps by developing and characterizing biodegradable chitosan films incorporating green-synthesized AgNPs@CHI or CuONPs@CHI films obtained from *Camellia sinensis* extract. The films were physicochemically characterized and tested against *Staphylococcus aureus*, *Escherichia coli*, and *Cutibacterium acnes*, with both AgNPs@CHI and CuONPs@CHI films showing bacteriostatic or bactericidal activity. Furthermore, cytotoxicity assays revealed selective toxicity toward healthy human cells, highlighting the therapeutic potential of these materials. Conventional therapies for acne vulgaris, while effective in many cases, face significant challenges. The prolonged use of topical and oral antibiotics, such as clindamycin and erythromycin, has led to the alarming emergence of antibiotic-resistant strains of *C. acnes*, compromising long-term treatment efficacy [11,12]. Furthermore, common keratolytic and antimicrobial agents like benzoyl peroxide and retinoids often cause undesirable side effects, including skin irritation, dryness, and photosensitivity, which can lead to poor patient adherence [11]. In this context, there is a pressing clinical need to develop alternative antimicrobial strategies that are not only effective against relevant pathogens but also selective, biocompatible, and capable of overcoming resistance mechanisms. The use of biopolymer-based nanocomposites such as those explored in this study represents a promising approach to fill this gap.

## 2. Results

### 2.1. Synthesis and Characterization of CuONPs and AgNPs Synthesized by Green Tea

**Figure 1:**
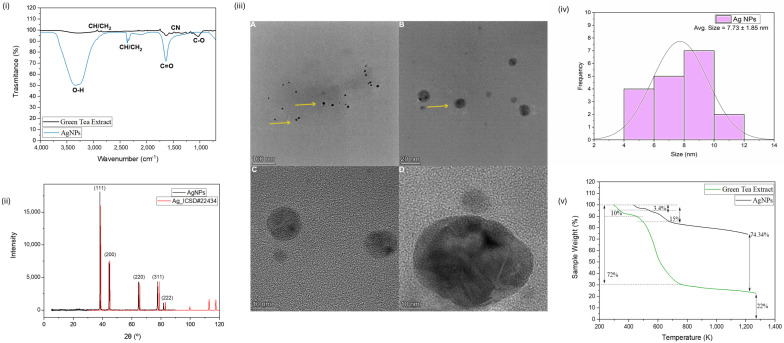
(**i**) FTIR spectra of AgNPs and green tea extract. (**ii**) XDR patterns for biogenic synthesized AgNPs and the pattern obtained from ICSD. (**iii**) HTEM micrograph of AgNPs and representative diameter distribution of AgNPs. The yellow arrows indicate the presence of the nanoparticles. (**iv**) Estimated size distribution of AgNPs. (**v**) TGA curves of green tea extract and AgNPs.

Biogenic CuONPs obtained with *Camellia sinensis* extract presented a core diameter of 9.11 ± 6.72 nm (HRTEM), hydrodynamic size of 78.0 ± 2.7 nm, PDI of 0.17 ± 0.04, and zeta potential of −26.5 ± 2.7 mV, while AgNPs showed a hydrodynamic diameter of 57.36 ± 3.9 nm, PDI of 0.32 ± 0.01, zeta potential of −37.5 ± 3.32 mV, and solid-state diameter of 7.73 ± 1.85 nm (Table 1, Figure 1i–v). CuONPs contained 59% organic matter, whereas AgNPs contained 25%, with higher residual (inorganic) mass for AgNPs (Table 1).

### 2.2. Characterization CHI Films, CuONPs@CHI Films, and AgNPs@CHI Films

**Figure 2:**
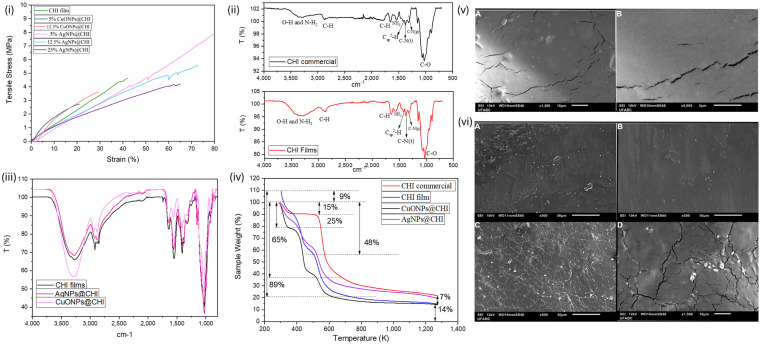
(**i**) Tensile stress–strain curves of chitosan-based films incorporated with different concentrations of green-synthesized AgNPs and CuONPs. (**ii**) FTIR spectra of commercial chitosan and chitosan films. (**iii**) FTIR spectra of chitosan films and chitosan and films incorporated with green-synthesized AgNPs and CuONPs. (**iv**) TGA curves obtained for commercial chitosan, CHI film, 25% AgNPs@CHI film, and 25% CuONPs@CHI film. (**v**) SEM images of CHI film (A) Top view (B) Bottom view. (**vi**) SEM images of 25% AgNPs@CHI film (A and B) and 25% CuONPs@CHI film (C and D).

Flexible, continuous chitosan (CHI) films were obtained by solvent casting with glycerol as the plasticizer. The control CHI film displayed a tensile strength of 4.4 ± 0.2 MPa and strain at break of 49.5 ± 6.5% (Figure 2i). Incorporation of 5% and 12.5% CuONPs reduced elongation (24.7 ± 3.8% and 21.5 ± 7.8%) and tensile strength (2.77 ± 0.11 and 3.15 ± 0.45 MPa), and the 25% CuONP sample was too brittle for testing (Figure 2i). AgNPs at 5% increased tensile strength to ~7.8 ± 0.5 MPa, with elongation of 94.1 ± 8.2%; higher AgNP loadings (12.5%, 25%) showed reduced but still higher values than the control (Figure 2i). FTIR spectra of commercial chitosan and CHI films exhibited characteristic O–H/N–H stretching (3500–3000 cm^−1^), C–H stretching at 2879 cm^−1^, amide-related bands at 1651 and 1540 cm^−1^, and C–O-region bands (1145–1000 cm^−1^) (Figure 2ii). After nanoparticle incorporation, the principal chitosan bands remained without significant shifts (Figure 2iii). Thermogravimetric analysis of CHI and nanocomposite films showed multi-stage mass losses with earlier onset for nanocomposites (Figure 2iv). SEM revealed a relatively smooth CHI surface with elongated streaks; AgNPs and CuONPs were distributed within these regions in their respective films (Figure 2v,vi).

### 2.3. Biological Essays

**Figure 3:**
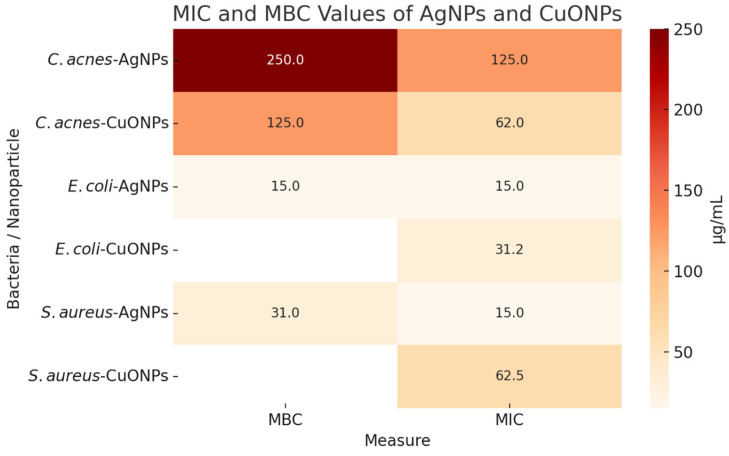
Minimum Inhibitory Concentration (MIC) and Minimum Bactericidal Concentration (MBC) values in µg/mL for green-synthesized silver (AgNPs) and copper oxide nanoparticles (CuONPs) tested against *Escherichia coli*, *Staphylococcus aureus*, and *Cutibacterium acnes*. Data are organized by nanoparticle type and bacterial strain. Blank cells indicate undetermined values (ND). CuONP data from [5].

**Figure 4:**
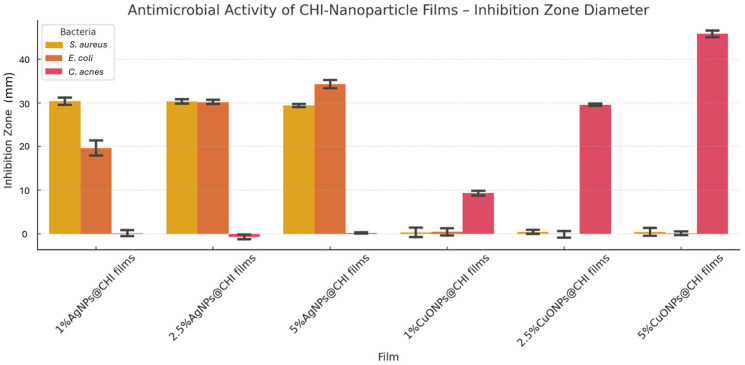
Inhibition zone diameters (mm) for chitosan-based films containing green-synthesized AgNPs or CuONPs at 1%, 2.5%, and 5% (*w/w*), tested against *E. coli*, *S. aureus*, and *C. acnes*. Data are presented as mean ± SD (n = 3). A two-way ANOVA revealed a significant interaction between nanoparticle type and bacterial strain (F(2,6) = 123.90, *p* < 0.0001, ηp^2^ = 0.9764). Tukey’s post hoc test confirmed that CuONPs were significantly more effective than AgNPs against *C. acnes* (*p* = 0.0002, 95% CI [21.23, 44.77]), while AgNPs outperformed CuONPs against *E. coli* (*p* = 0.0008) and *S. aureus* (*p* = 0.0035).

**Figure 5:**
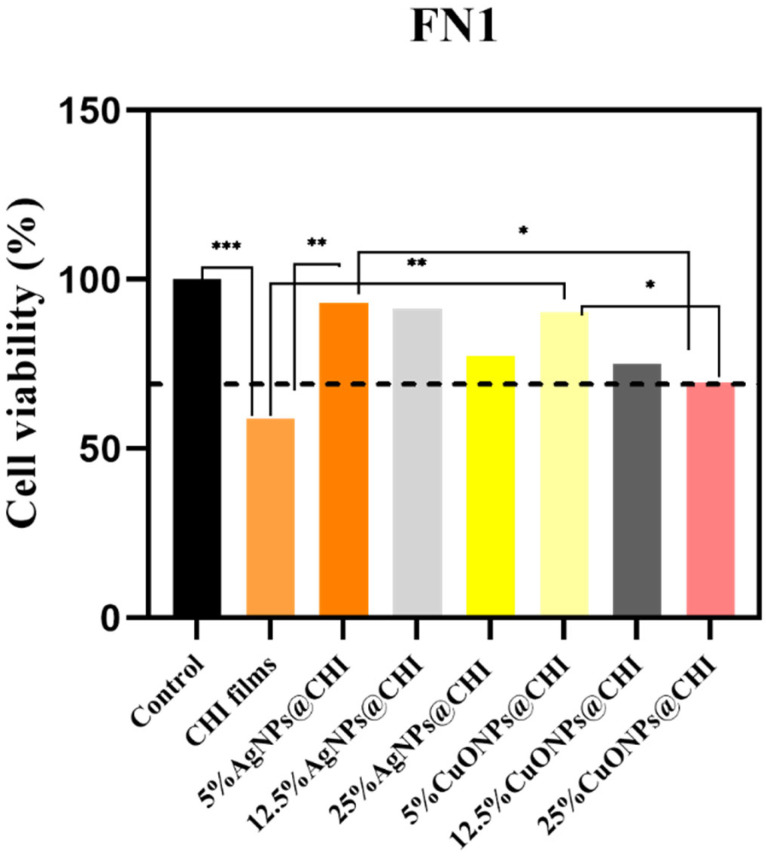
Viability (%) of FN1 human fibroblasts after 24 h exposure to chitosan-based films containing different concentrations of AgNPs or CuONPs. Data are shown as mean ± SD (n = 2). One-way ANOVA revealed a significant effect of treatment. Tukey’s post hoc test confirmed reduced viability in CHI films and high-concentration nanocomposites compared to the control (***p* < 0.05**, see Table S6). Asterisks indicate statistical significance compared with the control: * *p* < 0.05; ** *p* < 0.01; *** *p* < 0.001.

AgNPs exhibited MIC values of 15 µg/mL and MBCs of 15–31 µg/mL against *E. coli* and *S. aureus* (Figure 3). *C. acnes* showed higher MIC/MBC values (125 µg/mL and 250 µg/mL) for AgNPs (Figure 3). CuONPs showed increased activity toward *C. acnes* with an MIC of 62 µg/mL and MBC of 125 µg/mL (Figure 3). Pure CHI films produced no inhibition zones (Figure 4). At 5% loading, AgNPs@CHI films generated inhibition zones of 0.30 ± 0.01 cm for *S. aureus* and 0.35 ± 0.01 cm for *E. coli* (Table 2/Figure 4). CuONPs@CHI films (5%) produced an inhibition zone of 0.45 ± 0.01 cm against *C. acnes* (Table 2/Figure 4). A concentration-dependent increase in *E. coli* halo diameters was observed for AgNPs (20 mm at 1% to 35 mm at 5%) (Figure 4). All film groups maintained fibroblast (FN1) viability above the 70% ISO 10993-5 threshold (Figure 5), with a concentration-dependent decrease observed but remaining within non-cytotoxic limits (Figure 5).

## 3. Discussion

### 3.1. Synthesis and Characterization of CuONPs and AgNPs Synthesized by Green Tea

Data for CuONP characterization were presented in a recent publication [5]. Briefly, the biogenic CuONPs synthesized using *Camellia sinensis* (green tea) extract displayed an average core diameter of (9.11 ± 6.72) nm, as determined by HRTEM analysis. Dynamic light scattering measurements showed a hydrodynamic (number-average) size in the same nanometer scale, with a moderately low PDI of approximately 0.17 ± 0.04, indicating a narrow and relatively uniform size distribution. The zeta potential of the CuONPs was recorded at (−26.5 ± 2.7) mV, signifying good colloidal stability via electrostatic repulsion. Complementary XRD confirmed a monoclinic CuO crystalline phase, while FT-IR spectra detected functional groups of polyphenols and flavonoids from the tea extract, acting as reducing and capping agents. HRTEM images corroborated the roughly spherical morphology with slight aggregations, and TGA showed 59 % organic matter capping in CuONPs [5].

The nanoscale dimensions, surface functionalization, crystallinity, and stability of AgNPs synthesized via a biogenic route using green tea extract (Ag NPs) were confirmed through a combination of techniques. Dynamic light scattering (DLS) analysis revealed an average hydrodynamic diameter of (57.4 ± 3.9) nm, a moderate polydispersity index (PDI) of 0.32 ± 0.01, and a zeta potential of (−37.5 ± 3.3) mV. These values indicate good colloidal stability and a relatively narrow size distribution, consistent with successful nanoparticle synthesis. Compared to AgNPs synthesized from flaxseed extract, which showed a larger hydrodynamic size of 231.8 nm and a zeta potential of −44.5 mV [13], the Ag NPs displayed more favorable nanoscale dimensions. The discrepancy between the hydrodynamic diameter and the solid-state size observed in subsequent analyses is attributed to the hydration layer, ionic cloud, and surface-adsorbed phytochemicals surrounding the nanoparticles, as previously discussed [10,14].

The notable discrepancy between the hydrodynamic diameter measured by DLS and the solid-state core diameter observed by TEM is a common feature in biosynthesized nanoparticle systems and has important functional implications. The hydrodynamic size reflects the effective volume of the particle in aqueous suspension, including the hydration layer and the corona of capping phytochemicals. This larger size influences the transport and steric interactions of the nanoparticle in biological environments. On the other hand, the metallic core size (TEM) is directly related to the reactive surface area and the available reservoir for the release of metal ions, a key mechanism of antimicrobial activity. Therefore, these two measurements provide complementary information: the core size correlates with intrinsic potency, while the hydrodynamic size and surface chemistry (defined by the capping) govern bioavailability and interaction with the cellular target [15,16].

Fourier transform infrared (FTIR) spectroscopy further corroborated the presence of green tea phytoconstituents on the nanoparticle surface. As illustrated in Figure 1i, the spectra of the Ag NPs (blue line) and the green tea extract (black line) exhibited comparable profiles. The broad band at 3440 cm^−1^ corresponds to O–H stretching vibrations, while the signal at 2913 cm^−1^ is related to C–H and CH_2_ aliphatic vibrations, both of which are commonly attributed to catechin and other polyols in the extract [10]. The absorption band at 1630 cm^−1^ is associated with C=O stretching from ketones, quinones, carboxylic acids, and esters. A distinct peak at 1392 cm^−1^ indicates C–N stretching from aromatic amines, suggesting the presence of solubilized caffeine, while the peak at 1044 cm^−1^ corresponds to C–O–C stretching vibrations, supporting the presence of ether bonds [5]. Collectively, these findings confirm that the phytoconstituents of the *Camellia sinensis* extract, such as catechins, flavonoids, and other polyphenols, perform an essential dual function: they act as reducing agents responsible for the biosynthesis of the metallic Ag^0^ core and, simultaneously, as capping agents that adorn the nanoparticle surface, ensuring its colloidal stability and biocompatibility [17]. The crystalline structure of the Ag NPs was confirmed by X-ray diffraction (XRD). As presented in Figure 1ii, distinct diffraction peaks were observed at 2θ values of 38.26° (111), 44.44° (200), 64.57° (220), 77.41° (311), and 81.44° (222), which are characteristic of the face-centered cubic (FCC) structure of metallic silver (Ag^0^). Although the diffraction peaks are only slightly shifted—suggesting a minor expansion in the Ag lattice parameter—they correspond to those reported in the ICSD database (file #22434), and the absence of impurity peaks confirms the high purity of the synthesized nanoparticles [10,14].

High-transmission electron microscopy (HTEM) provided further insight into the morphology and solid-state size of AgNPs. As shown in Figure 1iii, the nanoparticles displayed a predominantly spherical morphology with no apparent aggregation, indicating efficient stabilization by the plant-derived capping agents. The particle size distribution (Figure 1iv), analyzed from the micrographs, revealed an average diameter of (7.73 ± 1.85) nm, which is substantially smaller than the hydrodynamic diameter obtained by DLS. This size difference, which is common in nanoparticle systems, results from the absence of solvent layers and agglomeration effects in the solid-state images. These findings confirm the successful biosynthesis of stable, nanoscale AgNPs, consistent with previous reports involving plant and bacterial routes [18,19].

The thermogravimetric analysis (TGA) profiles of the green tea extract and the synthesized AgNPs are shown in Figure 1v. The TGA curve of the pure green tea extract (green line) displays a significant weight loss of approximately 72% occurring between 320 and 800 K, which is attributed to the decomposition of volatile organic compounds and bioactive constituents such as polyphenols, flavonoids, and carbohydrates naturally present in the extract. A second, smaller weight loss of around 6% is observed at higher temperatures, likely due to further degradation of more stable residual organics or incomplete combustion products, leading to a final residual mass of approximately 22%. In contrast, the AgNP sample (black line) exhibits markedly improved thermal stability. The initial weight loss of ~10% below 450 K can be associated with the loss of adsorbed water and loosely bound organic molecules on the nanoparticle surface. Subsequent minor degradation steps occur between 450 and 800 K, with two distinguishable phases corresponding to losses of 3.45% and 15%, likely related to partial degradation of organic stabilizing agents bound to the silver surface. Overall, the AgNP sample retains approximately 74.34% of its weight up to 1200 K, indicating the presence of a significant inorganic residue attributed to silver content, along with enhanced thermal resistance imparted by the nanoparticle synthesis. These findings confirm that the formation of silver nanoparticles leads to significant enhancement in thermal stability, likely due to the incorporation and stabilization of bio-organic compounds onto the metallic surface, as well as the inherent thermal resistance of the metallic core. The increased residual mass in AgNPs also provides indirect evidence of the successful synthesis of silver nanoparticles and the partial encapsulation or functionalization with organic molecules from the green tea extract.

Table 1 above shows all the nanoparticle characteristics for this work. CuONPs and AgNPs, both synthesized using green tea extract, exhibit notable differences in physicochemical and thermal properties. CuONPs show greater organic content, larger size, and slight aggregation, while AgNPs are more monodisperse, colloidally stable, and thermally resistant. Both contain polyphenols and flavonoids but differ in crystallinity and morphological uniformity, indicating distinct behaviors for potential biomedical or antimicrobial applications.

### 3.2. Characterization of CHI Films, CuONPs@CHI Films, and AgNPs@CHI Films

The mechanical properties of polymeric films are critical determinants of their suitability for topical biomedical applications, including antimicrobial dressings, anti-acne patches, and cosmetic barrier films [20]. Such applications demand materials that exhibit a careful balance of moderate tensile strength, comfortable adherence to the skin, and adequate flexibility to conform to curved or mobile anatomical regions without cracking or delaminating [20,21].

In this study, chitosan films were obtained by solvent casting using aqueous acetic acid. Glycerol was added as a plasticizer and enhanced film elasticity, flexibility, and ductility, effectively mitigating the intrinsic brittleness of pure chitosan. Uniform and continuous films were obtained, with adequate flexibility promising the possibility of application on skin.

Tensile stress–strain analysis (Figure 2i) revealed that both the type and concentration of incorporated nanoparticles significantly influenced the films’ mechanical properties. The control chitosan film exhibited a tensile strength of 4.4 ± 0.2 MPa and strain at break of ~49.5 ± 6.5%. The incorporation of CuONPs at 5% and 12.5% resulted in films with reduced elongation at break (24.7 ± 3.8 and 21.5 ± 7.8%, respectively) and tensile strength (2.77 ± 0.11 and 3.15 ± 0.45 MPa) compared to the control, whereas the sample with 25% of CuONPs was too brittle to be tested. In contrast, the films prepared with AgNPs had improved mechanical properties. The 5% AgNPs@CHI exhibited the highest tensile strength (~7.8 ± 0.5 MPa) and elongation at break (94.1 ± 8.2%) among the samples, reflecting increased mechanical strength and ductility. Increasing the concentration of AgNPs (12.5 and 25 wt%) reduced the elongation at break (63.7 ± 12.0% and 46.6 ± 5.6%, respectively) and the tensile strength (4.9 ± 0.6 and 4.6 ± 0.4 MPa, respectively). However, even at the higher concentration of AgNPs, the films exhibited improved tensile properties relative to the control film.

The improved tensile properties observed with 5% AgNPs may be attributed to good nanoparticle dispersion and good interfacial interaction with chitosan, promoting a reinforcing effect [22]. Polyphenols on the AgNP surface can interact with chitosan through H bonds between the oxygen groups of the polyphenols and the hydroxyl or amine groups of chitosan and through electrostatic interactions between the negatively charged polyphenols and negatively charges chitosan that favor nanoparticle dispersion in the films. When the AgNP concentration increases, nanoparticles may agglomerate, and these agglomerates can act as stress concentration regions [22].

From this perspective, films containing intermediate concentrations of nanoparticles (e.g., 5 and 12.5% *w*/*w*) offer an optimal balance between mechanical strength and adaptability, making them particularly promising for flexible and functional topical biomaterials.

The FTIR spectra of commercial chitosan and chitosan films, as shown in Figure 2ii, revealed typical vibrational bands associated with chitosan’s functional groups. The broad absorption band between 3500 and 3000 cm^−1^ corresponds to O–H stretching and overlapping N–H stretching from amine groups, while the peak at 2879 cm^−1^ is associated with symmetric and asymmetric C–H stretching vibrations [23,24]. Additional bands at 1651 cm^−1^ and 1540 cm^−1^ reflect C=O stretching from acetoamide groups and N–H bending from primary amines, respectively, indicative of N-acetylglucosamine and glucosamine units. The presence of bands at 1418, 1370, and 1308 cm^−1^ was attributed to Csp^2^–H bending and C–N stretching of tertiary and primary amines. The absorptions at 1145, 1069, 1025, and 1000 cm^−1^ were assigned to various C–O bond vibrations, consistent with previous reports [24]. Slight shifts and band broadening observed in the film compared to the raw chitosan suggest interaction with acetic acid used during solubilization. As shown in Figure 2, SEM and TGA were employed to evaluate the structural, morphological, and thermal characteristics of the chitosan films before and after incorporation of metallic nanoparticles.

After the incorporation of silver and copper oxide nanoparticles, the FTIR spectrum of the composite films (Figure 2iii) maintained the principal absorption bands of chitosan, although with variations in intensity. No significant shifts in peak positions were detected, suggesting that the nanoparticles were physically embedded within the chitosan matrix without altering its chemical backbone [25].

Figure 2iv shows the TGA curves of commercial chitosan, chitosan film, and nanocomposite films with AgNPs and CuONPs. All samples demonstrated a three-stage mass loss profile. The initial loss (around 10% for powder and 21% for films) near 400 K was associated with moisture evaporation and hydrogen bond disruption. The second degradation phase at approximately 600 K involved decomposition of glycerol and biopolymeric components [25]. Notably, nanocomposite films exhibited slightly lower onset decomposition temperatures, likely due to the catalytic influence of metal ions or their interaction with the polymeric matrix. Above 700 K, further degradation and carbonization occurred, attributable to cleavage of glycosidic bonds and the release of small fatty acids and other degradation products [26]. Thermal analysis by TGA further elucidated the stability and composition of the films. Figure 2iv presents the thermogravimetric curves for Ag NPs and green tea extract. The thermal degradation of Ag NPs occurred in two main stages: A minor mass loss of approximately 3.4% near 370 K, attributed to volatile organic species, and a second, more significant loss of 15% around 600 K, corresponding to the decomposition of organic capping agents. These results, also observed in the green tea extract profile, reflect the organic content associated with phytochemical stabilization of the nanoparticles [10,27,28]. The residual mass was attributed to the silver core, indicating approximately 84.44% inorganic content in the AgNP formulation [29,30].

Morphological features of the films were examined using SEM. As shown in Figure 2v, the surface of the chitosan film without nanoparticles exhibited a relatively smooth topography with characteristic elongated streaks, likely formed due to non-uniform drying or molecular alignment of chitosan chains. Such textural features may arise from localized variations in the polymer concentration or from film casting conditions. Figure 2vi displays SEM images of chitosan films containing AgNPs (panels A and B) and CuONPs (panels C and D). The nanoparticles appeared to be evenly distributed but tended to accumulate within the streaked regions of the film. The embedding of nanoparticles within surface irregularities may influence the film’s physical and functional properties, including antimicrobial performance. Related studies have reported both homogeneous [25] and heterogeneous [31] nanoparticle distribution, depending on the synthesis method and compatibility with the chitosan matrix.

Altogether, these analyses confirm the successful incorporation of metallic nanoparticles into chitosan films, preserving the functional groups of the biopolymer, altering surface morphology in a controlled manner, and introducing thermal modifications consistent with composite behavior. These features support the potential application of the developed materials as stable, functional biomaterials for antimicrobial or packaging applications.

### 3.3. Antimicrobial Effects

#### 3.3.1. MIC and MBC Results for AgNPs and CuONPs

The MIC and MBC data (Figure 3) demonstrate notable differences in the antimicrobial efficacy of green-synthesized AgNPs and CuONPs. AgNPs were highly effective against *Escherichia coli* and *Staphylococcus aureus*, with MIC values of 15 µg/mL and MBCs ranging from 15 to 31 µg/mL. These low values are consistent with AgNPs’ well-documented bactericidal mechanisms, including membrane disruption and oxidative stress, which are particularly effective under aerobic conditions. However, *Cutibacterium acnes* exhibited marked resistance to AgNPs, with MIC and MBC values of 125 µg/mL and 250 µg/mL, respectively. Similar results were reported for AgNPs synthesized by *Cinnamomum tamala* [32], with MIC and MBC values of 12.5 μg/mL and 15 μg/mL, respectively, for *E. coli* and 10 μg/mL and 50 μg/mL, respectively, for *S. aureus*. AgNPs synthesized by *P. Hamala* extracts exhibited MIC and MBC values of 125 μg/mL against bacterium *Propionibacterium acnes* (current *C. acnes*) [33].

In contrast, CuONPs showed significantly improved activity against *C. acnes*, with MIC and MBC values reduced to 62 µg/mL and 125 µg/mL. This supports the hypothesis that CuONPs are more effective against anaerobic Gram-positive bacteria, likely due to their sustained ion release and action under low-oxygen conditions. Additionally, CuONPs demonstrated moderate activity against *E. coli* and *S. aureus*, with MICs of 31.25 µg/mL and 62.5 µg/mL, respectively, although MBCs were not reported in that study.

These results suggest that while AgNPs are preferred for aerobic infections, CuONPs may serve as a more suitable and targeted antimicrobial agent for the treatment of acne-related conditions where *C. acnes* is prevalent. A fundamental aspect governing the interaction between nanoparticles and bacteria is surface charge. Although we did not measure the zeta potential of the bacterial strains in this study, it is well-established that most bacteria, including *E. coli*, *S. aureus*, and *C. acnes*, possess a net-negative surface charge at physiological pH [34,35,36]. Our biogenic nanoparticles also display negative zeta potentials, which would initially suggest an electrostatic repulsion barrier. However, the potent observed antimicrobial activity—particularly the selective action of CuONPs against *C. acnes*—indicates that the interaction involves more complex mechanisms.

It is now recognized that nanoparticle–bacteria interactions can overcome electrostatic repulsion through several pathways: (i) the release of metal cations (Ag^+^ and Cu^2+^), which readily bind to negatively charged membrane components [37]; (ii) interaction with localized positively charged domains or membrane-associated proteins; and (iii) non-electrostatic forces such as van der Waals interactions and hydrogen bonding mediated by the polyphenol-rich capping agents [38]. Interestingly, although *C. acnes* also has a negatively charged surface, its unique membrane composition—including higher proportions of saturated fatty acids and specific lipids—may render it more permeable or susceptible to Cu^2+^ ions, especially under anaerobic or low-oxygen conditions typical of acne lesions. Additionally, the thicker organic coating of CuONPs may facilitate sustained Cu^2+^ release and longer residence time on the skin, enhancing selectivity. These multifactorial interactions ensure close contact and localized disruption, even in the presence of overall surface charge repulsion [39]. Together, these findings support the rational design of chitosan-based nanocomposites tailored to the physiological profile of the target pathogen. AgNPs@CHI films are more effective for general antimicrobial barriers, whereas CuONPs@CHI films offer promising specificity for dermatological applications, especially acne treatment.

#### 3.3.2. Agar Diffusion (Halo) Assay in Chitosan Films

The antimicrobial performance of AgNPs@CHI and CuONPs@CHI films was further assessed through agar diffusion assays. The results showed no inhibition zone for pure chitosan films, which can be attributed to the limited diffusion of chitosan in the agar medium—a finding consistent with previous reports in the literature [40]. However, all composite films demonstrated notable antimicrobial behavior.

The mechanism of the antimicrobial effect of chitosan is still being discussed. This bacteriostatic effect may be associated with the cationic nature of chitosan, resulting from the protonation of its amino groups under acidic conditions, allowing it to interact with the negatively charged bacterial cell surface. In Gram-negative bacteria, the bacteriostatic effect may be associated with the interaction of chitosan with negatively charged phosphate groups of polysaccharides located on the outer membrane of the bacterial cell [41,42]. Another factor to be discussed is the chelation of essential nutrients; chitosan has the ability to bind to essential metal ions such as calcium, magnesium, and iron, which are crucial for bacterial growth and metabolism. By chelating these essential nutrients, chitosan can inhibit bacterial growth [40,42,43,44].

For *S. aureus*, AgNPs@CHI films at all concentrations produced consistent inhibition zones, averaging 30 mm, whereas CuONPs@CHI films showed no visible halo (Figure 4). Statistical analysis (ANOVA and Tukey HSD, *p* < 0.05) confirmed that the inhibition halos of 5% of AgNPs in AgNPs@CHI films were significantly larger than those of 5% CuONPs in CuONPs@CHI, denoting a higher efficacy of 5% AgNPs in AgNPs@CHI against this Gram-positive strain under aerobic conditions.

In contrast, *E. coli* exhibited a gradient response to AgNPs, with halo diameters increasing from 20 mm (1%) to 35 mm (5%). CuONPs again showed no inhibition zone. Statistical comparisons validated the difference between AgNPs and CuONPs films, indicating that AgNPs are significantly more effective against *E. coli*, a Gram-negative bacterium. In contrast, Christopher et al., 2024 [45] used the well diffusion method to evaluate the antimicrobial activity of chitosan films with CuONPs in different concentrations against *E. coli* and *S. Aureus*; the results indicated that the inhibition efficiency of CuONPs was better against *S. aureus* compared to *E. coli*.

Interestingly, for *C. acnes*, only 5% of CuONPs in CuONPs@CHI films generated inhibition zones, with diameters increasing dose-dependently from 10 to 45 mm. The amount of 5% AgNPs in AgNPs@CHI films did not produce any measurable halos. The statistical analysis corroborated these findings, showing significant superiority of 5% CuONPs in CuONPs@CHI over 5% AgNPs in AgNPs@CHI against *C. acnes*. This behavior may be attributed to the unique interaction of copper ions with Gram-positive anaerobic cell walls and their efficient ROS-mediated damage, even in low-oxygen environments.

The greater efficacy of AgNPs@CHI films against *E. coli* (Gram-negative) compared to *S. aureus* (Gram-positive) can be attributed to the fundamental differences in bacterial cell-wall architecture. The cell wall of Gram-positive bacteria consists of a thick peptidoglycan layer (20–80 nm), which acts as a robust physical barrier, potentially limiting the penetration of nanoparticles and the diffusion of released Ag+ ions. In contrast, Gram-negative bacteria possess a much thinner peptidoglycan layer (2–3 nm), covered by an outer membrane. Although the outer membrane presents a challenge, the overall cell wall is considered less obstructive to the passage of small antimicrobial agents like AgNPs and their ions, facilitating access to the cytoplasmic membrane and intracellular components, which results in greater inhibitory activity [46,47,48,49]. Studies have shown that AgNPs rapidly disrupt the plasma membrane of Gram-negative bacteria, leading to depolarization, leakage of intracellular contents, and inhibition of cellular respiration, primarily due to the action of released Ag^+^ ions. The thick peptidoglycan in Gram-positive bacteria, however, provides a more substantial barrier, reducing the efficacy of AgNPs [46,47,48,49]. Additionally, the antibacterial activity of AgNPs is influenced by factors such as particle size, surface charge, and capping agents, but the fundamental cell-wall differences remain a key determinant of their relative efficacy [46,47,48,49].

These results provide a compelling case for the selective efficacy of metal-based nanoparticles based on bacterial structure and metabolic profile. While AgNPs offer broad-spectrum activity against aerobic pathogens, CuONPs emerge as a promising alternative specifically for anaerobic bacteria such as *C. acnes*. The use of chitosan as a biocompatible carrier further supports direct contact-mediated antimicrobial action, which is especially important for skin applications where diffusion may be limited.

The statistical validation reinforces the reliability and reproducibility of these effects (Table 2), positioning 5% CuONPs in CuONPs@CHI as a strategic formulation for acne-targeted biomaterials, while 5% AgNPs in AgNPs@CHI may be more suitable for general antimicrobial barriers. This dual-platform strategy highlights the versatility of biogenic nanoparticle–chitosan films in addressing diverse clinical and environmental microbial challenges.

### 3.4. Cytotoxicity Assay

The cell viability of the human fibroblast cell line(FN1) was evaluated after treatment with CHI films with AgNPs@CHI or CuONPs@CHI at concentrations of 1%, 2.5%, and 5% (*w*/*w* of AgNPs or CuONPs in the films), as represented in Figure 5. The untreated cells were used as a control group for 100% cell viability. According to ISO 10993-5 [46], a material can be considered cytotoxic when it reduces cell viability by more than 30%, as indicated by the dashed line shown in the graphs.

Although previous studies have reported chitosan films as non-cytotoxic, our results revealed that pure chitosan films reduced FN1 viability to 58.88 ± 4.31%, which is below the 70% threshold defined by ISO 10993-5, indicating moderate cytotoxicity. This reduction in FN1 cell viability at higher concentrations may be related to chitosan’s cationic interaction with mammalian cell membranes, which can disrupt membrane integrity similarly to its antifungal mechanism [2,3,50]. However, pure chitosan is rarely used in biomedical applications; it is typically modified or combined with other components to improve biocompatibility and mitigate potential cytotoxic effects, as highlighted in recent reviews [1,2]. As shown in Figure 5, for the human fibroblast cell line (FN1), cell viability decreased with increasing concentrations of metal nanoparticles (M-NPs) in the films. However, this reduction remained within acceptable limits of biocompatibility, indicating that the films were not cytotoxic, even at higher nanoparticle concentrations [51,52].

The choice of FN1 fibroblasts is especially relevant, as these cells are key components of the dermal layer and play a critical role in wound healing and skin regeneration. Thus, evaluating cytotoxicity in FN1 provides direct insight into the safety of these films for topical and dermatological applications, such as acne treatment. The observed concentration-dependent response corroborates previous findings that chitosan-based bio-nanocomposites may exhibit mild reductions in cell viability due to oxidative stress or nanoparticle release yet remain safe for use on healthy skin tissue when properly formulated.

The cytotoxic behavior of chitosan–nanoparticle composites depends on several factors, including their nanoparticle size, surface charge, degree of aggregation, and distribution within the polymer matrix [52]. These results align with earlier reports on the antimicrobial efficacy and selective bioactivity of metal nanoparticles embedded in chitosan, particularly when synthesized through green methods. Overall, their cytocompatibility with FN1 reinforces the potential of these biogenic nanocomposites for safe use in skin-targeted therapies.

## 4. Materials and Methods

### 4.1. Materials

Commercial green tea powder (*Camellia sinensis*—Shanghai, China, Batch: 64NH2306146WY) was obtained from Natural Corporation do Brazil, São Paulo, Brazil. Copper sulfate (CuSO_4_·5H_2_O), silver nitrate (AgNO_3_), phosphate-buffered saline (PBS), chitosan (low molecular weight, ≥75% deacetylated), dimethyl sulfoxide (DMSO), and 3-(4,5-dimethylthiazol-2-yl)-2,5-diphenyltetrazolium bromide (MTT) were purchased from Sigma-Aldrich, St. Louis, MO, USA. Sodium hydroxide (NaOH), acetic acid, and glycerol were acquired from Synth, Diadema, SP, Brazil. Mueller–Hinton agar (MH agar), Reinforced Clostridial Medium (RCM) agar, and GasPak EZ with indicator were obtained from BBL™, BD Diagnostics (Franklin Lakes, NJ, USA). Sodium chloride (NaCl), which was used for the preparation of sterile saline solution (0.85%), was also acquired from Synth.

### 4.2. AgNPs and CuONPs Synthesized by Green Tea

AgNPs and CuONPs were synthesized using *Camellia sinensis* (green tea) extract, following previously described protocols with minor modifications [6]. The extract was obtained by heating an aqueous suspension of *C. sinensis* powder (1.5 mg/mL for AgNPs; 2.0 mg/mL for CuONPs) at 75 °C for 30 min. The mixture was vacuum-filtered to remove solids, and the resulting extract was used immediately. For the synthesis of AgNPs, an aqueous solution of AgNO_3_ (0.1 mol/L) was added dropwise to the green tea extract under constant magnetic stirring. For CuONPs, an aqueous solution of CuSO_4_·5H_2_O (0.22 mmol/L) was similarly added to the extract. The pH of the mixtures was adjusted to 10.5 for AgNPs and 5.4 for CuONPs using NaOH (1 mol/L). Both suspensions were stirred thoroughly for 30 min at room temperature to allow for nanoparticle formation. The resulting nanoparticle suspensions were centrifuged at 1731 g for 10 min, and the precipitates were collected and washed six times with ultrapure water. Finally, the nanoparticles were freeze-dried using a benchtop freeze dryer and stored in airtight containers until further use [6,11].

### 4.3. Solvent Cast Chitosan Film Production

Chitosan-based films were obtained as described by Qiao et al., 2021 [23], with some modifications. For CHI film, 1 g of chitosan was added to 50 mL of 0.5% (*v/v*) acetic acid aqueous solution and 1 mL of glycerol. The solution was constantly stirred for approximately 5 h. The films were prepared by the solution casting method, where 5 mL of the chitosan solution was deposited in 8 cm diameter silicone molds and dried at room temperature (25 °C) for four days. The preparation of CHI films containing CuONPs or AgNPs, synthesized by green tea extract, was based on the protocol described Shankar et al., 2017 [25], with some modifications. For information about synthesis and characterization of CuONPs or AgNPs, see the Supplementary Materials.

First, 1 g of chitosan was added to 50 mL of 0.5% (*v/v*) acetic acid aqueous solution and 1 mL of glycerol. The solution was constantly stirred for approximately 5 h. A volume of 5 mL of chitosan solution was individually mixed with different concentrations (1.0, 2.5, and 5.0 mg/mL) of CuONPs or AgNPs dispersed in chitosan solution. Each final dispersion was constantly stirred for approximately 5 h. The films were prepared by the solution casting method, where 5 mL of each dispersion was deposited in 8 cm silicone molds and dried at room temperature (25 °C) for four days. This procedure led to the formation of chitosan nanocomposites incorporating silver (AgNPs@CHI films) or copper oxide nanoparticles (CuONPs@CHI films) at concentrations of 5, 12.5, and 25 wt%.

### 4.4. Characterization of CuONPs and AgNPs Synthesized by Green Tea

Dynamic light scattering (DLS) measurements were performed using a Zetasizer Nano ZS (Malvern Instruments Co., London, UK) at 25 °C to determine the hydrodynamic diameter, polydispersity index (PDI), and zeta potential (ζ) of AgNPs and CuONPs. Analyses were carried out in aqueous media using disposable folded capillary zeta cells (10 mm path length), with detection at a fixed angle of 173°. X-ray diffraction (XRD) analysis was conducted using a Bruker D8 Advance diffractometer (Bruker Corporation, Berlin, Germany) equipped with a Cu Kα radiation source and Bragg–Brentano geometry. Diffraction patterns were recorded at room temperature over a 2θ range from 20° to 120°, with a step size of 0.05° and a counting time of 5 s per step. Fourier transform infrared (FTIR) spectra were obtained using a UATR Two spectrophotometer (PerkinElmer, Shelton, CT, USA) operating in the range of 400 to 4000 cm^−1^ with a resolution of 4 cm^−1^ and 32 scans per sample. Measurements were performed in attenuated total reflectance (ATR) mode using a single-reflection diamond crystal, allowing for direct analysis of the dried nanoparticle powders. High-resolution transmission electron microscopy (HTEM) was used to assess nanoparticle morphology and solid-state size using a Talos F200X-G2 microscope (Thermo Fisher Scientific, Waltham, MA, USA). Samples were dispersed in isopropanol and sonicated for 10 min; then, a drop was deposited on a nickel TEM grid. Micrographs were analyzed using ImageJ.J3 software to estimate average particle diameters and morphology Thermal stability was assessed using a thermogravimetric analyzer (DSC-TGA SDT Q600 V20.9 Build 20, Federal University of São Paulo, UNIFESP, São Paulo, SP, Brazil). Approximately 5 mg of each film sample was placed in a standard platinum pan and heated from 300 to 1400 K at a rate of 10 K/min under an argon flow of 150 mL/min. The carbon content of the samples was determined based on the TGA curves.

### 4.5. Characterization of CHI Film, CuONPs@CHI Films, and AgNPs@CHI Films

Mechanical properties were measured using an Instron universal testing machine. Test specimens were cut into strips with dimensions of 15 mm × 4 mm (length × width), and tensile tests were performed at room temperature at a constant deformation rate of 20 mm/min. Each film was tested in triplicate, and the results are reported as average values.

FTIR analyses were conducted using a Perkin Elmer UATR Two spectrophotometer, operating in the range of 400 to 4000 cm^−1^, with 32 scans and a resolution of 4 cm^−1^. The measurements were carried out in attenuated total reflectance (ATR) mode using a single-reflection diamond crystal. Thermal stability was assessed using a thermogravimetric analyzer (DSC-TGA SDT Q600 V20.9 Build 20, Federal University of São Paulo, UNIFESP). Approximately 5 mg of each film sample was placed in a standard platinum pan and heated from 300 to 1400 K at a rate of 10 K/min under an argon flow of 150 mL/min. The carbon content of the samples was determined based on the TGA curves. The surface morphology of the films was examined using a field emission scanning electron microscope (FE-SEM, JSM-6701F, JEOL Ltd., Tokyo, Japan) at an accelerating voltage of 3 kV. Prior to imaging, all samples were sputter-coated with a thin layer of Au-Pt to ensure conductivity.

### 4.6. Antimicrobial Activity

#### 4.6.1. Determination of MIC and MBC Values for AgNPs and CuONPs

The minimum inhibitory concentration (MIC) was determined following the guidelines outlined in document M07 according by previous work [10] Antimicrobial concentrations were prepared in Mueller–Hinton (MH) broth, with the final bacterial concentration adjusted to 5 × 10^5^ CFU/mL. A positive control was prepared by adding the bacterial inoculum to MH broth without antimicrobial agents. The minimum bactericidal concentration (MBC) was determined by subculturing 10 μL from the MIC and higher concentrations onto MH agar plates. The MBC was defined as the lowest concentration that killed ≥ 99.9% of bacterial cells after 24 h of antimicrobial treatment, following the guidelines provided by the NCCLS (1999).

#### 4.6.2. Agar Diffusion Test for Films

The agar diffusion assay was used to evaluate the in vitro antibacterial activity of CHI films (control), CuONPs@CHI films (containing 1.0%, 2.5%, and 5.0% *w/w* of CuONPs), and AgNPs@CHI films (containing 1.0%, 2.5%, and 5.0% *w/w* of AgNPs). Square samples of each film (1 cm^2^) were cut and sterilized using ultraviolet (UV) light for 20 min on each side. The antibacterial test was carried out following the methodology adapted from the agar diffusion assay commonly used for antibiotic evaluation. The bacterial strains used were *Cutibacterium acnes* ATCC^®^ 11827™, *Staphylococcus aureus* ATCC^®^ 25923™, and *Escherichia coli* ATCC^®^ 25922™, obtained from the microorganism bank of the Basic and Applied Bacteriology Laboratory, Microbiology Department, State University of Londrina (Londrina, PR, Brazil). *S. aureus* and *E. coli* strains were cultured on Mueller–Hinton (MH) agar and adjusted to a MacFarland standard of 0.5 (1.5 × 10^8^ CFU/mL) in sterile saline solution (0.85% NaCl). Sterilized films were placed on agar plates previously inoculated with the corresponding bacteria and incubated for 24 h at 37 °C in a B.O.D. incubator (SS Scientific, Londrina, PR, Brazil). For *C. acnes*, RCM (Reinforced Clostridial Medium) agar was used, and plates were incubated under anaerobic conditions using GasPak EZ with indicator (BBL system) at 37 °C for 48 h. All experiments were performed in triplicate. After the incubation period, the presence or absence of inhibition zones was measured as an indication of antimicrobial activity.

### 4.7. Cytotoxicity Assay of CHI Film, AgNPs@CHI Films, and AgNPs@CHI Films

The cytotoxicity of CHI films, CuONPs@CHI films (with 1.0%, 2.5%, and 5.0% *w/w* of CuONPs), and AgNPs@CHI films (with 1.0%, 2.5%, and 5.0% *w/w* of AgNPs) was evaluated against non-tumoral human fibroblast cells (FN1), obtained from the Cell Bank of Instituto Butantan (São Paulo, SP, Brazil). Cells were cultured in RPMI 1640 medium supplemented with 10% fetal bovine serum (FBS), 100 U/mL penicillin, and 100 μg/mL streptomycin and incubated at 37 °C in a humidified atmosphere with 5% CO_2_. Cultures were maintained by subculturing every 2–3 days, and cell viability was periodically monitored using the trypan blue exclusion method. To evaluate the effects of the films, cells were seeded in 24-well plates at a density of 4 × 10^4^ cells/well and incubated for 24 h. After this period, the culture medium was replaced with 900 μL of fresh medium, and square film samples (approximately 14 mm^2^) of each group were added to the wells. The plates were then incubated for an additional 24 h under the same conditions. After incubation, the polymers and medium were removed, and the wells were washed three times with PBS. Then, 600 μL of FBS-free medium containing MTT solution (0.3 mg/mL) was added to each well, and the plates were incubated for 2 h at 37 °C. Subsequently, the MTT solution was removed, and the formed formazan crystals were dissolved in 500 μL of dimethyl sulfoxide (DMSO). Absorbance was measured at 570 nm using a microplate reader (Asys Expert Plus, Biochrom, Cambridge, UK). Cells without treatment were used as the control group (100% cell viability). All experiments were performed in quadruplicate, and the results are expressed as mean ± standard deviation (SD).

### 4.8. Statistical Analysis

Data were analyzed using Origin (2018) and GraphPad Prism 9 software. Statistical significance was determined by two-way (halo) and one-way (cytotoxicity) ANOVA followed by Tukey’s multiple-comparisons tests. *p* values < 0.05 were considered significant. The results are presented as mean ± standard deviation (SD).

## 5. Conclusions

This study demonstrates the successful development of chitosan-based films incorporating green-synthesized silver and copper oxide nanoparticles, designed for antimicrobial action on the skin. AgNPs@CHI films exhibited strong and broad-spectrum activity against *Staphylococcus aureus* and *Escherichia coli*, with inhibition zones up to 0.35 cm and MIC values of 15 µg/mL. In contrast, CuONPs@CHI films showed selective and superior efficacy against *Cutibacterium acnes*, with halo diameters reaching 0.45 cm, an MIC of 62 µg/mL, and an MBC of 125 µg/mL. Pure chitosan films showed no inhibition zones, underscoring the essential role of the nanoparticles. All formulations maintained acceptable cytocompatibility, with fibroblast (FN1) viability consistently above the 70% threshold defined by ISO 10993-5. Although this study establishes the selective antimicrobial efficacy and cytocompatibility of the films, future work should investigate other functional properties critical for dermatological use, such as water vapor permeability and swelling behavior, to fully evaluate their potential as functional skin dressings and acne treatment patches. Additionally, while the 24 h cytotoxicity meets ISO standards, longer-term studies and evaluations in more complex skin models will be necessary to confirm safety for prolonged topical use.

Taken together, these findings highlight the multifunctionality of the developed biocomposites. AgNPs@CHI films are effective antimicrobial coatings for general skin protection, while CuONPs@CHI films offer targeted action against anaerobic pathogens, which is particularly relevant for acne treatment. Importantly, both systems combine high antimicrobial efficacy with pathogen-specific selectivity and biocompatibility. This unique profile positions these materials as strong candidates for next-generation dermatological therapies.

## Figures and Tables

**Table 1:** Comparative physicochemical characterization of biogenic copper oxide nanoparticles (CuONPs) and silver nanoparticles (AgNPs) synthesized using *Camellia sinensis* (green tea) extract. Data include the core and hydrodynamic size, colloidal stability (zeta potential and PDI), crystalline structure (XRD), organic functional groups (FTIR), morphology (HTEM), and thermal behavior (TGA).

Parameter	CuONPs *	AgNPs
Core diameter (TEM)/nm	9.11 ± 6.72	7.73 ± 1.85
Hydrodynamic size (DLS)/nm	78.0 ± 2.7	57.36 ± 3.9
Polydispersity index (PDI)	0.17 ± 0.04	0.32 ± 0.01
Zeta potential (mV)	−26.5 ± 2.7	−37.5 ± 3.32
Crystalline structure (XRD)	CuO (ICSD #16025)	Ag^0^ (ICSD #22434)
FTIR functional groups	Polyphenols and flavonoids	Polyphenols and flavonoids
Morphology (HTEM)	Roughly spherical and slight aggregation	Spherical, with no apparent aggregation
Organic content (TGA)	59% organic matter	25% organic matter
Thermal stability (TGA)	Less stable than AgNPs; higher organic loss	High thermal stability; ~74% residual mass at 1200 K

* Results from [5].

**Table 2:** Summary of antimicrobial activity (halo inhibition) of chitosan films containing AgNPs or CuONPs against three bacterial strains. Values represent the highest mean ± SD for each nanoparticle at 5%. Statistical differences were assessed using two-way ANOVA followed by Tukey’s post hoc test (*p* < 0.05).

Bacteria	5% AgNPs in AgNPs@CHI Film Inhibition Zone (cm)	5% CuONPs in CuONPs@CHI Film Inhibition Zone (cm)	Statistical Comparison	Interpretation
***S. aureus***	0.30 ± 0.01	0.00 ± 0.00	AgNPs > CuONPs (*p* < 0.05)	AgNPs: highly effective against Gram-positive aerobes
***E. coli***	0.35 ± 0.01	0.00 ± 0.00	AgNPs > CuONPs (*p* < 0.05)	AgNPs: effective against Gram-negative bacteria
***C. acnes***	0.00 ± 0.00	0.45 ± 0.01	CuONPs > AgNPs (*p* < 0.05)	CuONPs: selectively effective against anaerobic *C. acnes*

## Supplementary Materials

The following supporting information can be downloaded at: https://www.mdpi.com/article/10.3390/antibiotics14090891/s1, **Figure S1.** Halo diffusion test of CHI films against *E. coli* and *S. aureus*. **Figure S2.** Halo diffusion test of CHI films with (A) 5% CuONPs@CHI, (B) 12.5% CuONPs@CHI, (C) 25% CuONPs@CHI, (D) 5% AgNPs@CHI, (E) 12.5% AgNPs@CHI, and (F) 25% AgNPs@CHI against *S. aureus*. **Figure S3.** Halo diffusion test of CHI films with (A) 5% CuONPs@CHI, (B) 12.5% CuONPs@CHI, (C) 25% CuONPs@CHI, (D) 5% AgNPs@CHI, (E) 12.5% AgNPs@CHI, and (F) 25% AgNPs@CHI against *E. coli*. **Figure S4.** Halo diffusion test of CHI films with (A) 5% CuONPs@CHI, (B) 12.5% CuONPs@CHI, (C) 25% CuONPs@CHI, (D) 5% AgNPs@CHI, (E) 12.5% AgNPs@CHI, and (F) 25% AgNPs@CHI against *C. acnes*. **Table S1.** Effect and statistic data for MIC/MBC essay. **Table S2.** Pairwise significant data for MIC/MBC assays. **Table S3.** Triplicate data extracted from Figures S2–S4, analyzed using ImageJ. **Table S4.** Effect and statistical data for halo diffusion assays. **Table S5.** Pairwise significant data for halo diffusion assays. **Table S6.** Viability (%) of FN1 human fibroblasts after 24 h exposure to chitosan-based films containing different concentrations of AgNPs or CuONPs.

## Abbreviations

The following abbreviations are used in this manuscript:

**Abbreviation**	**Meaning**
AgNPs	Silver nanoparticles
AgNPs@CHI film(s)	Chitosan film(s) containing silver nanoparticles
ATR	Attenuated Total Reflectance
ATCC	American Type Culture Collection
CHI films	Chitosan films
CFU	Colony-Forming Unit
CuONPs	Copper oxide nanoparticles
CuONPs@CHI film(s)	Chitosan film(s) containing copper oxide nanoparticles
DLS	Dynamic Light Scattering
FTIR	Fourier Transform Infrared
HRTEM	High-Resolution Transmission Electron Microscopy
ISO	International Organization for Standardization
MBC	Minimum Bactericidal Concentration
MIC	Minimum Inhibitory Concentration
PBS	Phosphate-Buffered Saline
PDI	Polydispersity Index
SEM	Scanning Electron Microscopy
TEM	Transmission Electron Microscopy
TGA	Thermogravimetric Analysis
