# Supplementary material for: Selective Antimicrobial Chitosan Films Incorporating Green-Synthesized Silver and Copper Oxide Nanoparticles for Acne Treatment
Source: Antibiotics (Basel). 2025 Sep 3;14(9):891. doi: 10.3390/antibiotics14090891 (PMC12466391; doi:10.3390/antibiotics14090891)
Supplement: Supplementary file 1 [file antibiotics-14-00891-s001.zip › antibiotics-3804216-supplementary.pdf]

Selective Antimicrobial Chitosan Films Incorporating Green-Synthesized Silver and Copper Oxide Nanoparticles for Acne Treatment

Roberta Albino dos Reis [1,*], Carolina C. de Freitas [1], Leonardo Longuini da Silva [1], Laura Pierobão Monteiro [2], Gerson Nakazato [2], Mathilde Champeau [1], Ricardo A. Galdino da Silva [3] and Amedea Barozzi Seabra [1,*]

[1] Centro de Ciências Naturais e Humanas (CCNH), Universidade Federal do ABC (UFABC),
Santo André 09210-580, SP, Brazil; carolina.freitas@ufabc.edu.br (C.C.d.F.); mathilde.champeau@ufabc.edu.br (M.C.)

[2] Laboratory of Basic and Applied Bacteriology, Department of Microbiology, Center of Biological Sciences, State University of Londrina, Londrina 86057-970, PR, Brazil; laura.pierobao@uel.br (L.P.M.);
gnakazato@uel.br (G.N.)

[3] Instituto de Ciências Ambientais, Químicas e Farmacêuticas, Universidade Federal de São Paulo,
Diadema 09913-030, SP, Brazil; galdino.ricardo@unifesp.br

* Correspondence: roberta.reis@ufabc.edu.br (R.A.d.R.); amedea.seabra@ufabc.edu.br (A.B.S.)

Table S1. Effect and statistic data for MIC/MBC essay

Effect	df	Sum of Squares	F value	p-value	Partial Eta Squared (ηp^2)
C(Group)	7	2728.436	18.13009	0.000257	0.940701
Residual	8	171.9911			0.5

Table S2. *Significant data for* MIC/MBC essay

Group 1	Group 2	Mean Difference	95% CI Lower	95% CI Upper	Adjusted p-value	Significant
12.5%AgNPs@CHI	12.5%CuONPs@CHI	-16.2479	-34.5958	2.0999	0.0891	FALSE
12.5%AgNPs@CHI	25%AgNPs@CHI	-14.0203	-32.3681	4.3275	0.1645	FALSE
12.5%AgNPs@CHI	25%CuONPs@CHI	-21.7791	-40.1269	-3.4313	0.02	TRUE
12.5%AgNPs@CHI	5%AgNPs@CHI	1.7359	-16.6119	20.0837	0.9999	FALSE
12.5%AgNPs@CHI	5%CuONPs@CHI	-0.99	-19.3378	17.3579	1	FALSE
12.5%AgNPs@CHI	CHI films	-32.611	-50.9588	-14.2632	0.0016	TRUE
12.5%AgNPs@CHI	Control	8.5134	-9.8344	26.8613	0.6175	FALSE

12.5%CuONPs@CHI	25%AgNPs@CHI	2.2276	-16.1202	20.5755	0.9995	FALSE
12.5%CuONPs@CHI	25%CuONPs@CHI	-5.5312	-23.879	12.8167	0.9134	FALSE
12.5%CuONPs@CHI	5%AgNPs@CHI	17.9839	-0.364	36.3317	0.0552	FALSE
12.5%CuONPs@CHI	5%CuONPs@CHI	15.258	-3.0899	33.6058	0.1172	FALSE
12.5%CuONPs@CHI	CHI films	-16.3631	-34.7109	1.9848	0.0863	FALSE
12.5%CuONPs@CHI	Control	24.7614	6.4136	43.1092	0.0094	TRUE
25%AgNPs@CHI	25%CuONPs@CHI	-7.7588	-26.1066	10.589	0.7032	FALSE
25%AgNPs@CHI	5%AgNPs@CHI	15.7562	-2.5916	34.104	0.1021	FALSE
25%AgNPs@CHI	5%CuONPs@CHI	13.0303	-5.3175	31.3782	0.2147	FALSE
25%AgNPs@CHI	CHI films	-18.5907	-36.9385	-0.2429	0.0468	TRUE
25%AgNPs@CHI	Control	22.5337	4.1859	40.8816	0.0164	TRUE
25%CuONPs@CHI	5%AgNPs@CHI	23.515	5.1672	41.8628	0.0128	TRUE
25%CuONPs@CHI	5%CuONPs@CHI	20.7891	2.4413	39.137	0.0259	TRUE
25%CuONPs@CHI	CHI films	-10.8319	-29.1797	7.5159	0.3755	FALSE
25%CuONPs@CHI	Control	30.2925	11.9447	48.6404	0.0026	TRUE
5%AgNPs@CHI	5%CuONPs@CHI	-2.7259	-21.0737	15.6219	0.9981	FALSE
5%AgNPs@CHI	CHI films	-34.3469	-52.6947	-15.9991	0.0011	TRUE
5%AgNPs@CHI	Control	6.7775	-11.5703	25.1254	0.808	FALSE
5%CuONPs@CHI	CHI films	-31.621	-49.9689	-13.2732	0.0019	TRUE
5%CuONPs@CHI	Control	9.5034	-8.8444	27.8512	0.5073	FALSE
CHI films	Control	41.1244	22.7766	59.4723	0.0003	TRUE

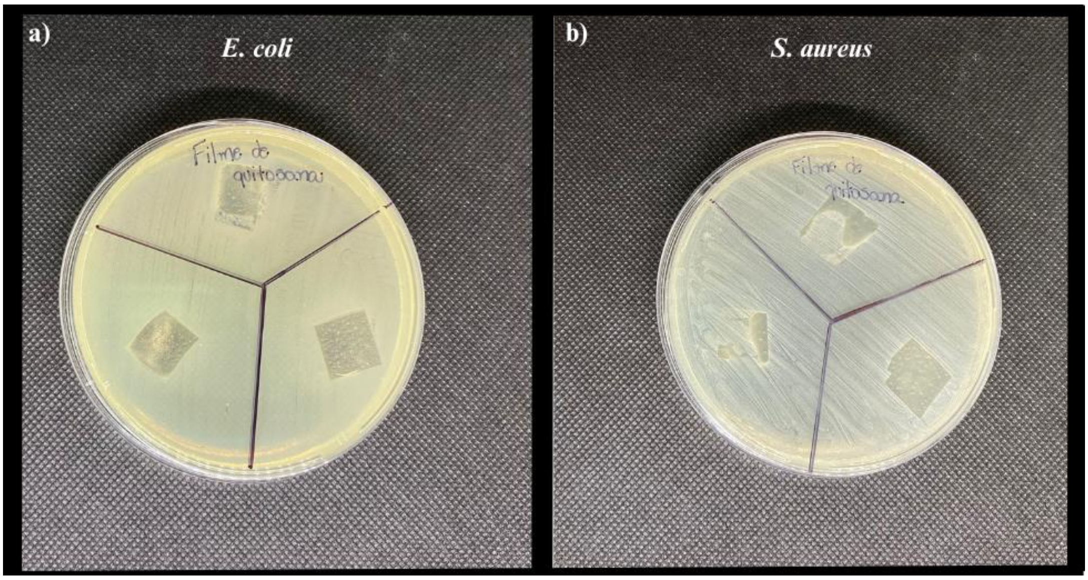

Figure S1 Halo diffusion test of CHI films against a) *E coli* and b) *S. aureus*

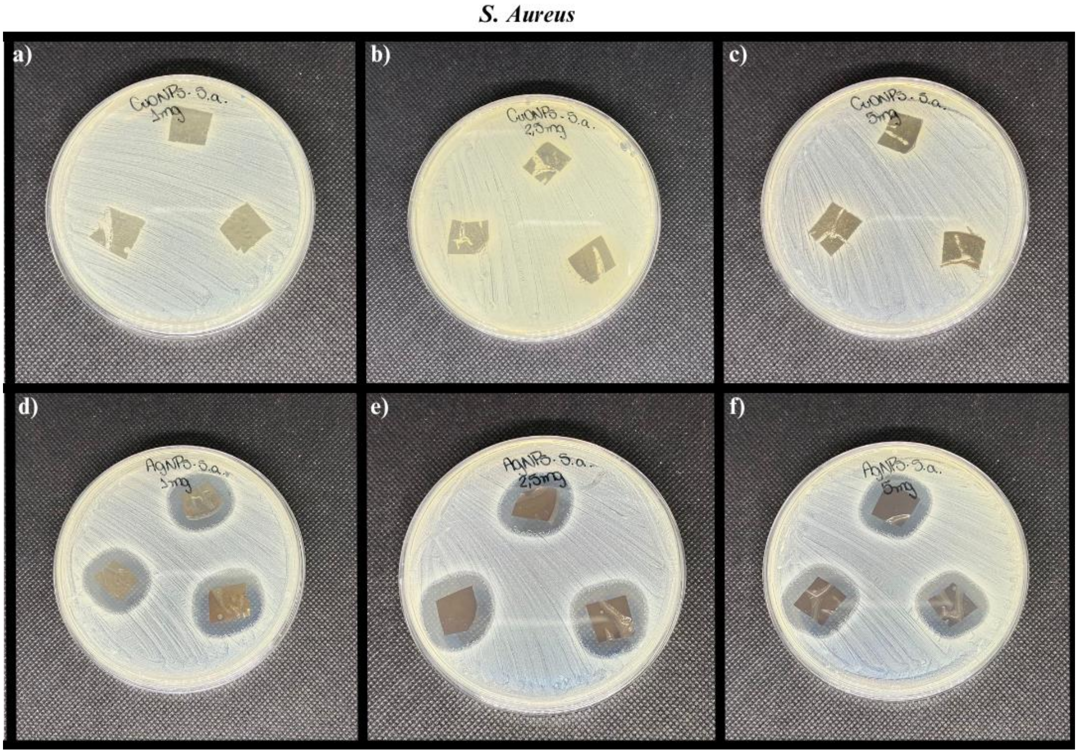

Figure S2. Halo diffusion test of CHI films with A) 5%CuONPs@CHI B) 12.5%CuONPs@CHI; C) 25%CuONPs@CHI; D) 5%AgNPs@CHI; E) 12.5%AgNPs@CHI and F) 25%AgNPs@CHI against *S. aureus*.

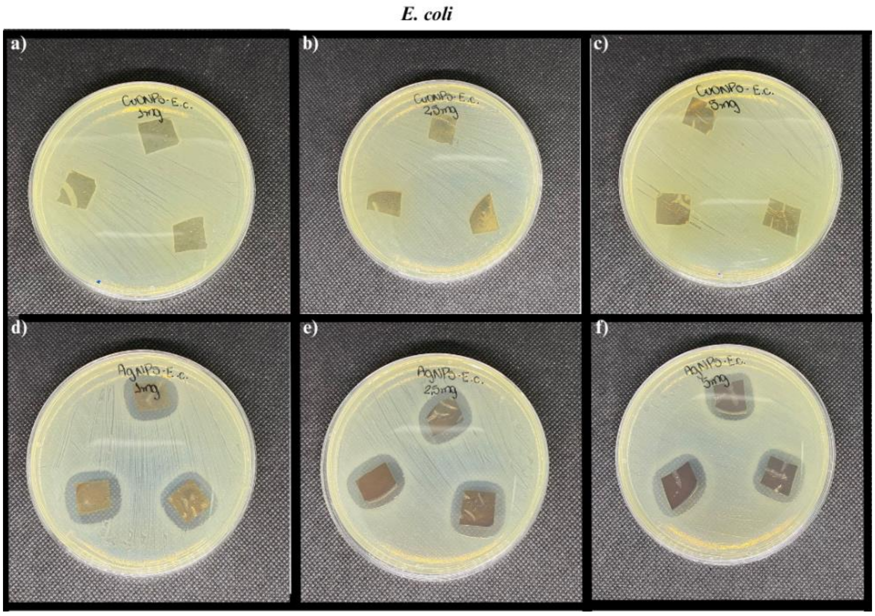

Figure S3. Halo diffusion test of CHI films with A) 5%CuONPs@CHI B) 12.5%CuONPs@CHI; C) 25%CuONPs@CHI; D) 5%AgNPs@CHI; E) 12.5%AgNPs@CHI and F) 25%AgNPs@CHI against *E. coli*.

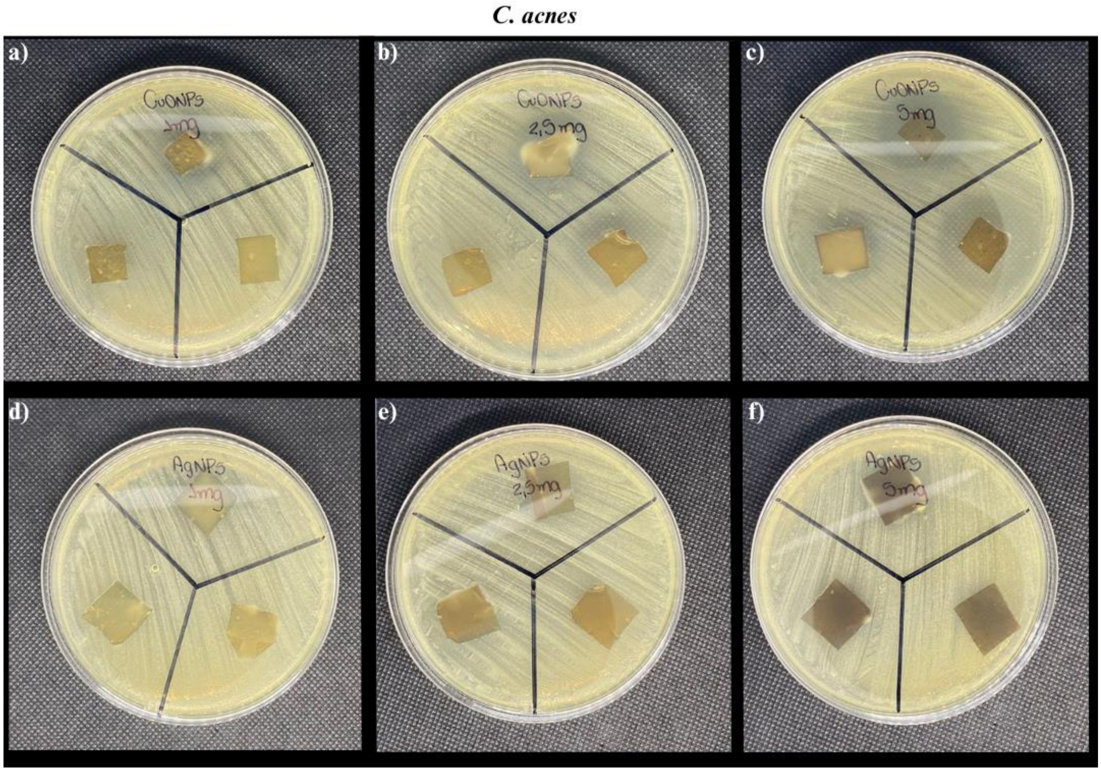

Figure S4. Halo diffusion test of CHI films with A) 5%CuONPs@CHI B) 12.5%CuONPs@CHI; C) 25%CuONPs@CHI; D) 5%AgNPs@CHI; E) 12.5%AgNPs@CHI and F) 25%AgNPs@CHI against *C. acnes*.

Table S3. Triplicate data from Figures S 2-4. Data was analised by ImageJ.

Bacteria	Film	Halo (cm)
S. aureus	5%AgNPs@CHI	0.31
S. aureus	5%AgNPs@CHI	0.31
S. aureus	5%AgNPs@CHI	0.29
S. aureus	12.5%AgNPs@CHI	0.30
S. aureus	12.5%AgNPs@CHI	0.30
S. aureus	12.5%AgNPs@CHI	0.31
S. aureus	25%AgNPs@CHI	0.30
S. aureus	25%AgNPs@CHI	0.30
S. aureus	25%AgNPs@CHI	0.29
S. aureus	5%CuONPs@CHI	-0.01
S. aureus	5%CuONPs@CHI	0.01
S. aureus	5%CuONPs@CHI	0.01
S. aureus	12.5%CuONPs@CHI	0.00
S. aureus	12.5%CuONPs@CHI	0.01
S. aureus	12.5%CuONPs@CHI	0.00
S. aureus	25%CuONPs@CHI	-0.01
S. aureus	25%CuONPs@CHI	0.00
S. aureus	25%CuONPs@CHI	0.02
E. coli	5%AgNPs@CHI	0.20
E. coli	5%AgNPs@CHI	0.22
E. coli	5%AgNPs@CHI	0.17
E. coli	12.5%AgNPs@CHI	0.31
E. coli	12.5%AgNPs@CHI	0.30
E. coli	12.5%AgNPs@CHI	0.30
E. coli	25%AgNPs@CHI	0.35
E. coli	25%AgNPs@CHI	0.33
E. coli	25%AgNPs@CHI	0.35
E. coli	5%CuONPs@CHI	0.00
E. coli	5%CuONPs@CHI	0.01
E. coli	5%CuONPs@CHI	-0.01
E. coli	12.5%CuONPs@CHI	-0.01
E. coli	12.5%CuONPs@CHI	-0.01
E. coli	12.5%CuONPs@CHI	0.01
E. coli	25%CuONPs@CHI	0.00
E. coli	25%CuONPs@CHI	-0.01
E. coli	25%CuONPs@CHI	0.01
C. acnes	5%AgNPs@CHI	0.00
C. acnes	5%AgNPs@CHI	0.01
C. acnes	5%AgNPs@CHI	-0.01
C. acnes	12.5%AgNPs@CHI	0.00
C. acnes	12.5%AgNPs@CHI	0.00

C. acnes	12.5%AgNPs@CHI	-0.01
C. acnes	25%AgNPs@CHI	0.00
C. acnes	25%AgNPs@CHI	0.00
C. acnes	25%AgNPs@CHI	0.00
C. acnes	5%CuONPs@CHI	0.10
C. acnes	5%CuONPs@CHI	0.09
C. acnes	5%CuONPs@CHI	0.10
C. acnes	12.5%CuONPs@CHI	0.30
C. acnes	12.5%CuONPs@CHI	0.29
C. acnes	12.5%CuONPs@CHI	0.30
C. acnes	25%CuONPs@CHI	0.45
C. acnes	25%CuONPs@CHI	0.47
C. acnes	25%CuONPs@CHI	0.45

Table S4. Effect and statistic data for halo essay

Effect	df	Sum of Squares	F value	p-value	Partial Eta Squared (ηp²)
C(NP_Type)	1	70.08333	8.009524	0.029953	0.57172
C(Strain)	2	188.1667	10.75238	0.010381	0.781856
C(NP_Type):C(Strain)	2	2168.167	123.8952	1.32E-05	0.976358
Residual	6	52.5			0.5

Table S5. Significant data for halo essay

Group 1	Group 2	Mean Difference	95% CI Lower	95% CI Upper	Adjusted p-value	Significant
Ag - C_acnes	*Ag - E_coli*	22	10.2275	33.7725	0.0024	true
Ag - C_acnes	*Ag - S_aureus*	18	6.2275	29.7725	0.0068	true
Ag - C_acnes	*CuO - C_acnes*	33	21.2275	44.7725	0.0002	true
Ag - C_acnes	*CuO - E_coli*	-5	-16.7725	6.7725	0.5802	false
Ag - C_acnes	*CuO - S_aureus*	-2.5	-14.2725	9.2725	0.9472	false
Ag - E_coli	*Ag - S_aureus*	-4	-15.7725	7.7725	0.7518	false
Ag - E_coli	*CuO - C_acnes*	11	-0.7725	22.7725	0.0661	false
Ag - E_coli	*CuO - E_coli*	-27	-38.7725	-15.2275	0.0008	true

Ag - E_coli	*CuO - S_aureus*	-24.5	-36.2725	-12.7275	0.0013	true
Ag - S_aureus	*CuO - C_acnes*	15	3.2275	26.7725	0.0168	true
Ag - S_aureus	*CuO - E_coli*	-23	-34.7725	-11.2275	0.0019	true
Ag - S_aureus	*CuO - S_aureus*	-20.5	-32.2725	-8.7275	0.0035	true
CuO - C_acnes	*CuO - E_coli*	-38	-49.7725	-26.2275	0.0001	true
CuO - C_acnes	*CuO - S_aureus*	-35.5	-47.2725	-23.7275	0.0002	true
CuO - E_coli	*CuO - S_aureus*	2.5	-9.2725	14.2725	0.9472	true

Table S6. Viability (%) of FN1 human fibroblasts after 24 h exposure to chitosan-based films containing different concentrations of AgNPs or CuONPs. Data are shown as mean ± SD (n = 2).

**Group**	**Mean Viability (%)**	**SD (n=2)**
Control	100	0
CHI films	58.87555	4.674050771
5%AgNPs@CHI	93.22247	3.254969952
12.5%AgNPs@CHI	91.486565	7.751157565
25%AgNPs@CHI	77.46626	0.983910876
5%CuONPs@CHI	90.49659	4.75231156
12.5%CuONPs@CHI	75.238615	2.930456217
25%CuONPs@CHI	69.70746	6.879612518
